# Multimodality imaging of Xp11.2 translocation/TFE3 gene fusion associated with renal cell carcinoma: a case report

**DOI:** 10.3389/fmed.2023.1266630

**Published:** 2023-09-19

**Authors:** Wenpeng Huang, Yushuo Peng, Yongbai Zhang, Yongkang Qiu, Yi Liu, Aixiang Wang, Lei Kang

**Affiliations:** ^1^Department of Nuclear Medicine, Peking University First Hospital, Beijing, China; ^2^Department of Urology, Peking University First Hospital, Beijing, China; ^3^Institute of Urology, Peking University, Beijing, China; ^4^National Urological Cancer Center, Beijing, China

**Keywords:** translocation renal cell carcinoma, Xp11.2, ultrasound, computed tomography, magnetic resonance imaging, ^18^F-FDG, PET/CT

## Abstract

**Background:**

Xp11.2 translocation/TFE3 gene fusion associated with renal cell carcinoma (Xp11.2 RCC) exhibits unique biological characteristics and is associated with an increased incidence of tumor thrombosis, lymph node metastasis, and advanced disease stages. Multimodality imaging, including US, contrast-enhanced CT, multi-parametric MRI, and ^18^F-FDG PET/CT plays a crucial role in the preoperative diagnosis and differentiation of renal tumors.

**Case report:**

A 15-year-old female presented with lumbar pain worsened, and developed persistent painless hematuria. The CT attenuation values of the scan without contrast, corticomedullary phase, nephrographic phase, and delayed phases were 35 HU, 83 HU, 82 HU, and 75 HU, respectively. The solid component of the mass displayed heterogeneous marked enhancement. Furthermore, MRU indicated that the lesion involved the cortical medulla and infringed on the renal sinus fat. The lesion appeared isosignal in T1WI, slightly low signal in T2WI, and slightly high signal in DWI. The degree of enhancement in the three phases of enhancement scan was lower than that in the renal parenchyma, and hemorrhage and necrosis were observed within the internal part of the lesion. To further clarify the staging, the patient underwent ^18^F-FDG PET/CT. PET/CT images showed multiple irregular occupancies in the right kidney with unclear borders, showing a heterogeneous increase in ^18^F-FDG uptake, with SUVmax values ranging from 2.3 to 5.2 in the routine imaging phase (60 min post-injection), compared to SUVmax values ranging from 2.8 to 6.9 in the delayed imaging phase (160 min post-injection). Additionally, multiple enlarged and fused lymph nodes were observed in the medial part of the right kidney and the retroperitoneum, exhibiting a heterogeneous increase in ^18^F-FDG uptake, with SUVmax values ranging from 4.1 to 8.7 in the routine imaging phase, compared to SUVmax values ranging from 4.4 to 9.1 in the delayed imaging phase. The postoperative pathology, immunohistochemistry, and molecular analysis of histiocytes were consistent with a diagnosis of Xp11.2 RCC. One month after surgery, enhanced-CT examination of the patient revealed lung metastasis, peritoneal metastasis, and multiple lymph node metastases throughout the body, with an overall survival of 16 months.

**Conclusion:**

Xp11.2 RCC exhibits unique biological characteristics and is associated with an increased incidence of tumor thrombosis, lymph node metastasis, and advanced disease stages. Long-term follow-up is essential to monitor the likelihood of recurrence and metastasis. ^18^F-FDG PET/CT examination can comprehensively visualize the lesion’s location and extent, providing a basis for clinical tumor staging and aiding in treatment monitoring and follow-up. To address the limitations of FDG, the utilization of specific tracers designed for RCC or tracers that are not excreted via the urinary system would be ideal. Further advancements in molecular imaging technologies and the development of novel tracers hold great promise in advancing the diagnosis and management of RCC, ultimately contributing to better patient outcomes and overall disease management.

## Introduction

Annually, malignant kidney tumors are diagnosed in 3–15 individuals per 100,000 (1). Apart from the well-known clear cell cancers, which constitute 80–85% of all renal cell cancer (RCC) cases, the 2016 World Health Organization (WHO) classification introduced new molecular-driven histotypes. These include MiTF family translocation carcinoma, succinate dehydrogenase (SDH)-deficient RCC, and hereditary leiomyomatosis and RCC syndrome-associated RCC (2). One infrequent RCC subtype is RCC associated with Xp11.2 translocation/TFE3 gene fusion (Xp11.2 RCC) (3, 4), which has been integrated into the MiT family translocation RCC category. The significance of molecular data in enhancing kidney tumor classification was affirmed in the latest WHO classification. This new classification introduced a category of molecularly defined RCC, encompassing TFE3-rearranged RCC, TFEB-rearranged and TFEB-amplified RCC, FH-deficient RCC, SDH-deficient RCC, ALK-rearranged RCC, ELOC (formerly TCEB1)-mutated RCC, and SMARCB1 (INI1)-deficient RCC (5, 6). For TFE3-rearranged RCC, the identification of gene rearrangement through FISH assays or RNA-sequencing stands as the gold standard for confirming diagnosis (7, 8). Due to distinct translocation genes implicated, varying morphology, and notably different behavior, TFE3-rearranged RCC are currently recognized as distinct entities. Additionally, Xp11.2 RCC has been documented to display a more aggressive behavior when compared to other RCC subtypes, exhibiting a propensity for distant and lymphatic metastases, ultimately leading to a poorer prognosis (9, 10).

To enhance understanding of this rare neoplasm, we present a case study detailing the multimodal imaging manifestations of a patient with Xp11.2 RCC. Following surgical resection of the tumor, the patient experienced rapid disease progression, developing pulmonary metastases, peritoneal metastases, and multiple lymph node metastases throughout the body, ultimately resulting in an overall survival of 16 months.

## Case presentation

A 15-year-old female presented with right lumbar pain, which began 2 years ago and was left untreated. Two months ago, the patient’s lumbar pain worsened, and she developed persistent painless hematuria. On admission, physical examination revealed a palpable hard mass in the right upper abdomen. Laboratory tests showed elevated alpha-fetoprotein levels (18.32 ng/mL).

**Figure 1:**
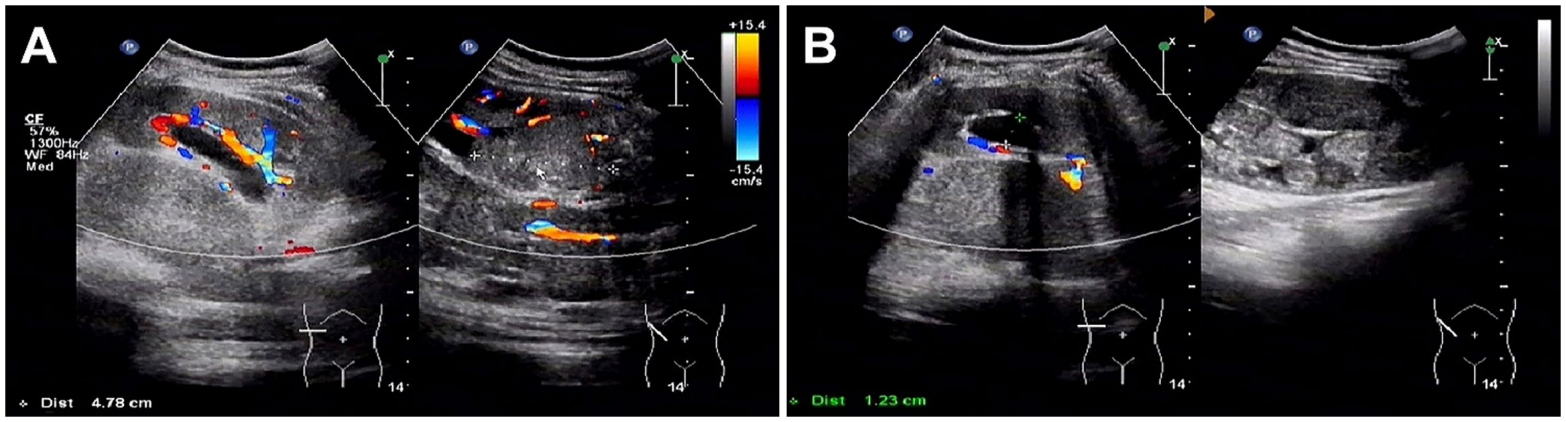
Ultrasound images of Xp11.2 translocation/TFE3 gene fusion associated with renal cell carcinoma (Xp11.2 RCC). **(A)** A highly heterogeneous hypoechoic mass in the right subrenal region with an irregular, lobulated morphology. The mass encroached on the renal sinus and protruded outward from the kidney. A class I blood flow signal was observed within and around the mass. **(B)** Multiple enlarged lymph nodes were detected adjacent to the right renal hilum, in proximity to the inferior vena cava, in the right adrenal region, and near the abdominal aorta.

**Figure 2:**
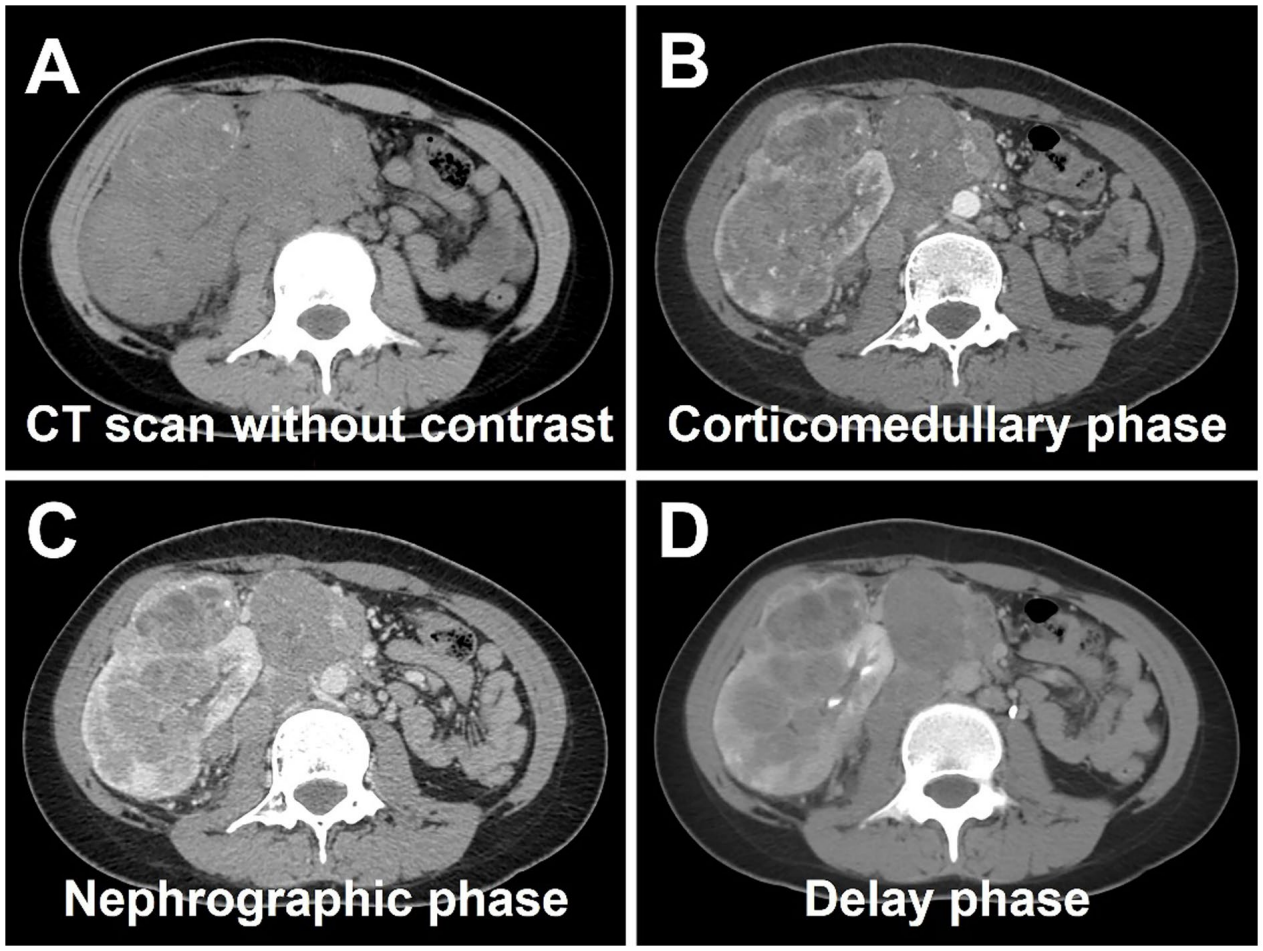
Computed tomography (CT) images of Xp11.2 RCC. **(A)** The transverse image shows a right renal mass measuring approximately 5.2 cm × 7.1 cm × 8.8 cm with multiple calcifications. **(B–D)** The CT attenuation values of corticomedullary phase, nephrographic phase, and delayed phases were 83 HU, 82 HU, and 75 HU, respectively. The solid component of the mass displayed heterogeneous marked enhancement, with areas of hypodense necrosis observed within. Several fused lymph nodes were identified in the medial right kidney and retroperitoneum, showing the same density and enhancement pattern as the right intrarenal lesion.

**Figure 3:**
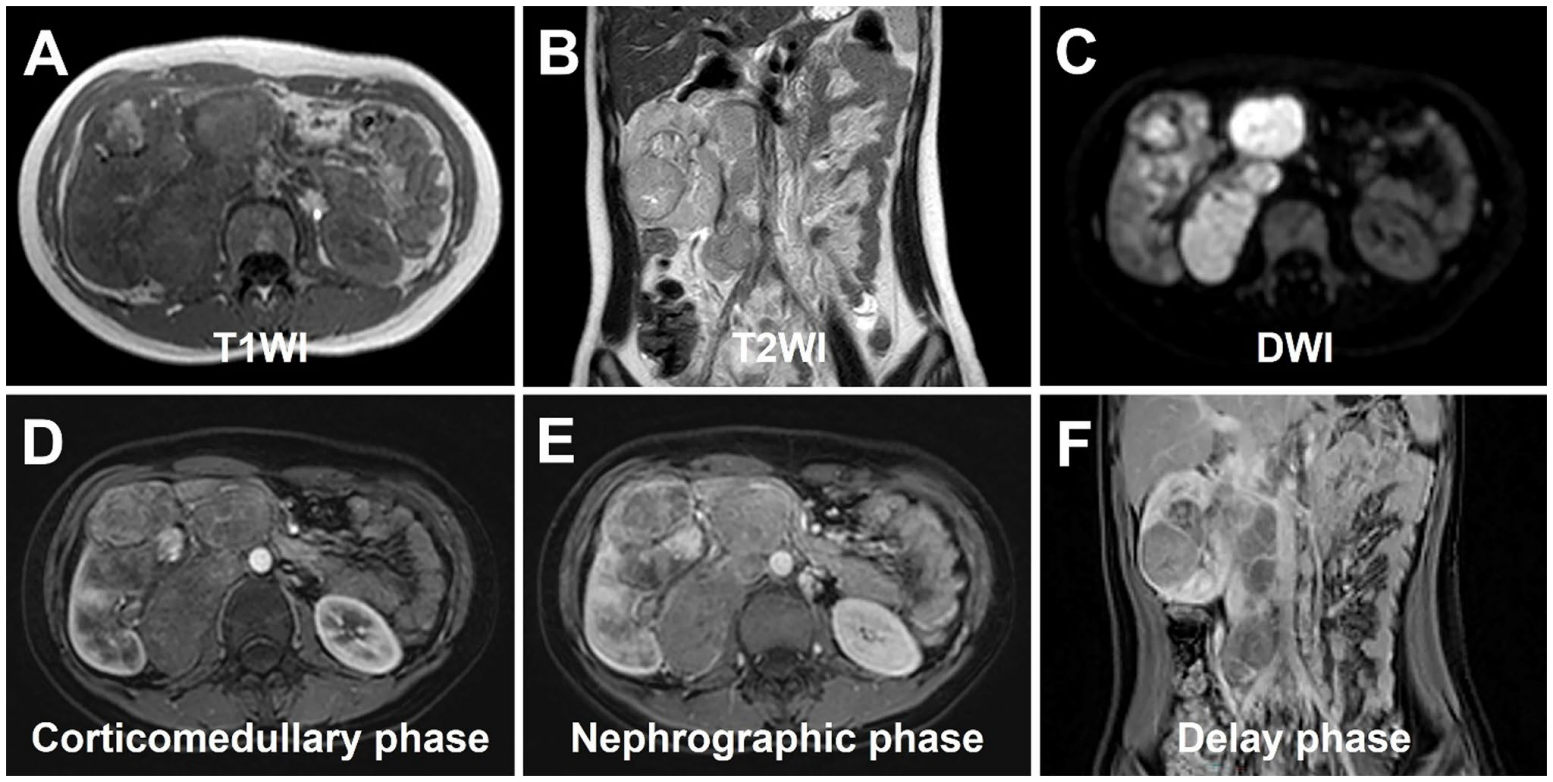
magnetic resonance urography (MRU) of Xp11.2 RCC. **(A)** T1WI transverse image shows a heterogeneous isosignal lesion. **(B)** T2WI transverse image shows a heterogeneous slightly low signal lesion. **(C)** DWI transverse image shows a slightly high signal lesion. **(D–F)** The degree of enhancement in the three phases of enhancement scan was lower than that in the renal parenchyma, and hemorrhage and necrosis were observed within the internal part of the lesion. Additionally, pseudocoated membrane was observed around the lesion.

Abdominal ultrasound was performed, revealing a highly heterogeneous hypoechoic mass in the lower right abdomen with an irregular, lobulated morphology. The mass encroached on the renal sinus and protruded outward from the kidney. A class I blood flow signal was observed within and around the mass (Figure 1A). Additionally, multiple enlarged lymph nodes were detected adjacent to the right renal hilum, in proximity to the inferior vena cava, in the right adrenal region, and near the abdominal aorta. These lymph nodes were fused together and exhibited uneven echogenicity (Figure 1B). CT examination revealed a right renal mass measuring approximately 5.2 cm × 7.1 cm × 8.8 cm (AP × LR × SI) with multiple calcifications. The CT attenuation values of the scan without contrast, corticomedullary phase, nephrographic phase, and delayed phases were 35 HU, 83 HU, 82 HU, and 75 HU, respectively. The solid component of the mass displayed heterogeneous marked enhancement, with areas of hypodense necrosis observed within. Additionally, several fused lymph nodes were identified in the medial right kidney and retroperitoneum, showing the same density and enhancement pattern as the right intrarenal lesion (Figure 2). Furthermore, magnetic resonance urography (MRU) indicated that the lesion was located in the ventral part of the middle and lower pole of the right kidney. The lesion involved the cortical medulla and infringed on the renal sinus fat. The lesion appeared isosignal in T1WI, slightly low signal in T2WI, and slightly high signal in DWI (Figures 3A–C). The degree of enhancement in the three phases of enhancement scan was lower than that in the renal parenchyma, and hemorrhage and necrosis were observed within the internal part of the lesion. Additionally, pseudocoated membrane was observed around the lesion (Figures 3D–F).

**Figure 4:**
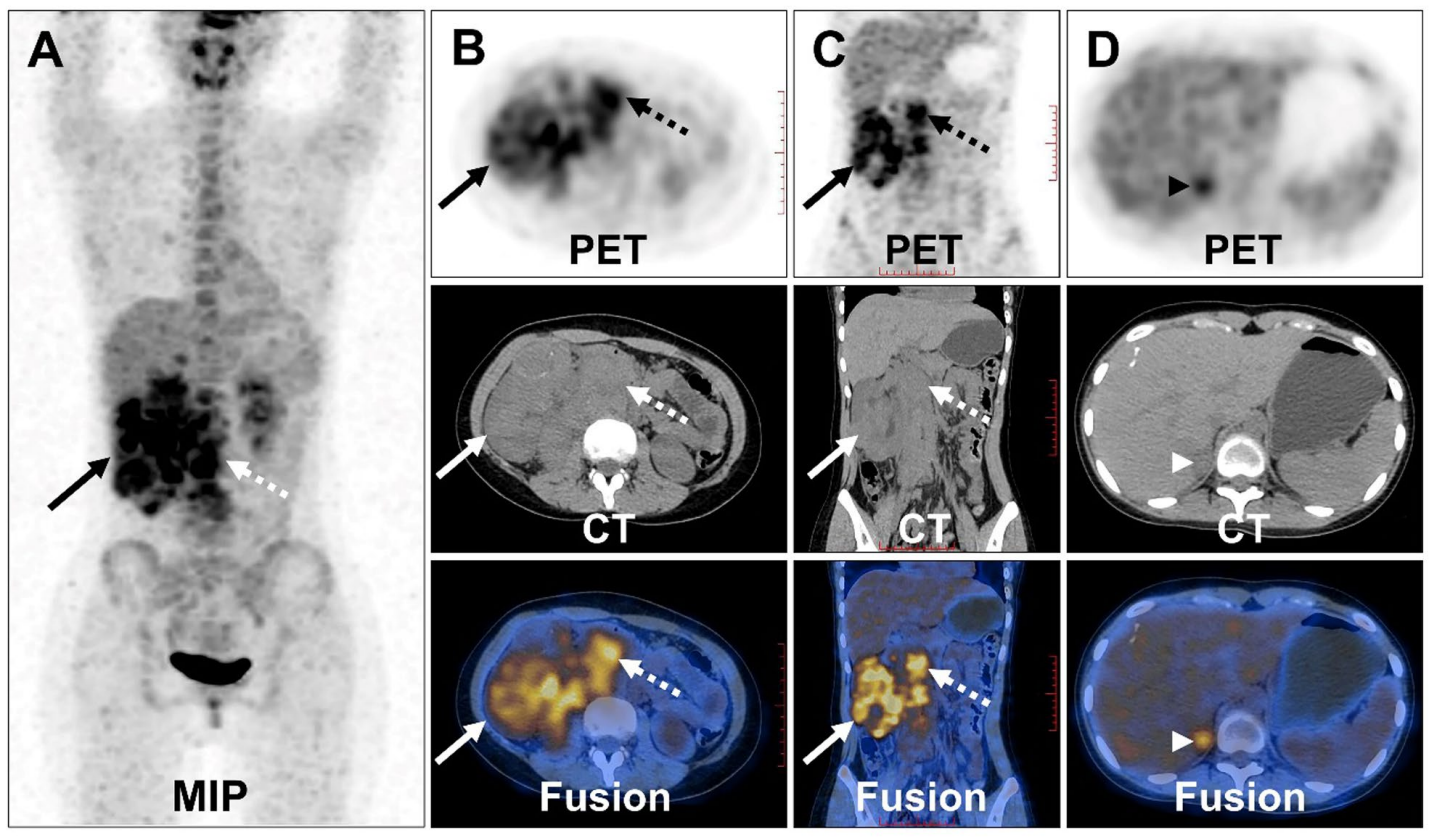
^18^F-FDG PET/CT images of Xp11.2 RCC. **(A)** The anteroposterior 3-dimensional maximum intensity projection image (MIP) demonstrated increased metabolic activity in the right kidney (long arrows) and retroperitoneal lymph nodes (dashed arrows). **(B,C)** Transverse images and coronal images showed multiple irregular occupancies in the right kidney with unclear borders, showing a heterogeneous increase in FDG uptake, with SUVmax values ranging from 2.3 to 5.2. Multiple enlarged and fused lymph nodes were observed in the medial part of the right kidney and the retroperitoneum, exhibiting a heterogeneous increase in FDG uptake, with SUVmax values ranging from 4.1 to 8.7. **(D)** Transverse images showed a soft tissue density nodule of the right adrenal gland measuring approximately 1.2 cm × 2.0 cm (arrowheads), with increased FDG uptake (SUVmax = 4.4).

Compression and forward displacement of the right renal vein and inferior vena cava were present, with no evidence of tumor thrombus in the vein. To further clarify the staging, the patient underwent ^18^F-FDG PET/CT to evaluate the lesions. PET/CT images showed multiple irregular occupancies in the right kidney with unclear borders, showing a heterogeneous increase in ^18^F-FDG uptake, with SUVmax values ranging from 2.3 to 5.2 in the routine imaging phase (60 min post-injection), compared to SUVmax values ranging from 2.8 to 6.9 in the delayed imaging phase (160 min post-injection). Additionally, multiple enlarged and fused lymph nodes were observed in the medial part of the right kidney and the retroperitoneum, exhibiting a heterogeneous increase in ^18^F-FDG uptake, with SUVmax values ranging from 4.1 to 8.7 in the routine imaging phase, compared to SUVmax values ranging from 4.4 to 9.1 in the delayed imaging phase. The lesions were poorly demarcated from the right renal artery, right ureter, lower vena cava, and the head of the pancreas, and they were not well demarcated from the right renal artery. Furthermore, the lesion exhibited poor demarcation from the right kidney, right renal artery, right ureter, inferior vena cava, abdominal aorta, and the head of the pancreas, with the right kidney being displaced laterally due to compression, resulting in dilated hydronephrosis of the right renal pelvis (Figures 4A–C). In the right adrenal gland, a soft tissue density nodule measuring approximately 1.2 cm × 2.0 cm was visible, showing increased ^18^F-FDG uptake with an SUVmax of 4.4 (Figure 4D), compared to SUVmax of 4.9 measured during delayed imaging. Based on the diagnostic imaging, the patient was considered to have nephroblastoma with concurrent retroperitoneal lymph node and right adrenal gland metastases.

**Figure 5:**
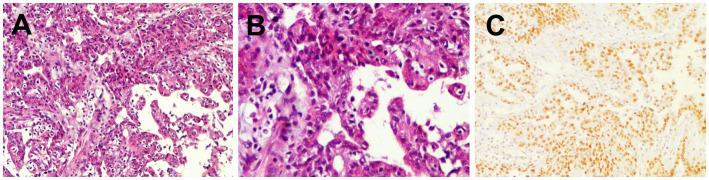
Histopathological and immunohistochemical images of Xp11.2 RCC. **(A,B)** Hematoxylin–eosin (HE) staining (magnification ×100 and 200) showed papillary structures composed of epithelioid clear cells, eosinophils, and gravel bodies. **(C)** Immunohistochemistry showed that the tumor cells were positive for TFE-3 (magnification ×100).

The patient underwent surgical resection of the right renal lesion and lymph node dissection. Postoperative pathologic microscopy revealed papillary structures composed of epithelioid clear cells, eosinophils, and gravel bodies (Figures 5A,B). Immunohistochemistry showed positive expression of TFE-3 (Figure 5C), HMB45, P504S, CD10, Vimentin, GATA-3, and AE1/AE3. The molecular analysis of histiocytes further revealed the *TFE3* gene disruption. The above findings were consistent with the diagnosis of Xp11.2 RCC. One month after surgery, enhanced-CT examination of the patient revealed lung metastasis, peritoneal metastasis, and multiple lymph node metastases throughout the body, with an overall survival of 16 months.

## Discussion

Xp11.2 RCC, a rare subtype of RCC, predominantly affects children rather than adults, accounting for 20–40% of pediatric RCC cases and 1–1.6% of RCC cases in adults (11). Commonly, patients with Xp11.2/TFE3 RCC present with nonspecific symptoms such as abdominal pain, flank mass, and gross hematuria (12). Despite advancements in research, the underlying mechanism of Xp11.2 RCC remains elusive. Some researchers have postulated that the abnormal upregulation of the HGF/MET pathway is closely related to the uncontrolled proliferation, metastasis, and invasion of tumor cells, which occurs when the TFE3 fusion protein binds with the MET promoter, leading to increased MET protein expression (13). Additionally, the inactivation of the FLCN tumor suppressor gene has been suggested to elevate the transcriptional activity of the TFE3 protein, contributing to tumorigenesis (14). According to Argani et al. (15), Xp11.2-RCC results from the fusion of the TFE3 gene with one of five different genes: ASPL (17q25), PRCC (1q21), PSF (1q34), NonO (Xq12), and CLTC (17q23), characterized by chromosomal rearrangements including t(X;17)(p11.2;q25), t(X;1)(p11.2;q21), t(X;1)(p11.2;p34), t(X;17)(p11.2;q23), and inv.(X)(p11.2;q12), respectively (16).

Pathological diagnosis of Xp11.2 RCC is challenging due to its heterogeneous features, characterized by prominent eosinophilic cytoplasm and a papillary growth pattern (17–19). The overexpression of TFE3 protein resulting from translocations makes immunohistochemistry (IHC) assay the most commonly used diagnostic technique in clinical practice (20). However, false negatives and positives may occur. Currently, fluorescence *in situ* hybridization (FISH) detection is considered the most reliable method for diagnosing Xp11.2 RCC (13).

Multimodality imaging, including conventional ultrasound (US), contrast-enhanced US (CEUS), computed tomography (CT), and multi-parametric magnetic resonance imaging (MRI), plays a crucial role in the preoperative diagnosis and differentiation of renal tumors (21). While most Xp11.2 RCCs are located in the renal medullary tissue or in both the medullary and cortical tissues within the kidney’s contour, some may breach the renal envelope, leading to alterations in the renal contour and manifesting as sizeable masses (22). These tumors often exhibit heterogeneity attributed to cystic degeneration, necrosis, or hemorrhage.

CEUS offers numerous advantages, including noninvasiveness, real-time imaging capabilities, and the accurate assessment of renal tumor characteristics and vascularity. In a study conducted by Ma et al. (23), fast wash-out patterns were identified as the primary features of Xp11.2 RCC. Furthermore, Wei et al. (24) investigated the differentiation between Xp11.2 RCC and clear cell type renal cell carcinoma (ccRCC) using CEUS and found that Xp11.2 RCC exhibited lower peak enhancement compared to ccRCC. CT examination offers the advantage of high spatial resolution and the ability to detect and recognize calcifications in the evaluation of RCC. Previous studies have emphasized the utility of CT characteristics and dynamic contrast-enhanced patterns in distinguishing Xp11.2 RCC from clear cell RCC (ccRCC) (25, 26). In a comparative study by He et al. (26), which included 20 cases of Xp11.2 RCC and 21 cases of renal clear cell carcinoma, the tumor-to-cortex attenuation ratio in the corticomedullary phase, with a cutoff value of less than 0.62, demonstrated a sensitivity of 90.0% and a specificity of 92.9% for differentiating Xp11.2 RCC from ccRCC. CT characteristics and dynamic contrast-enhanced patterns, along with the index, were effective in distinguishing Xp11.2 RCC from ccRCC. MRI examination plays an essential role in the evaluation of RCC, offering advantages such as superior soft tissue contrast, multi-planar imaging capabilities, and the ability to assess tumor vascularity and invasion, thereby facilitating accurate diagnosis and treatment planning. In terms of MRI signal features, Xp11.2 RCC typically appears hyper- to isointense on T1WI and heterogeneously hypointense on T2WI, with restricted diffusion observed on DWI. However, when the tumor involves hemorrhage and lipid, the signal can be hybrid in both T1WI and T2WI. Tumor necrosis and hemorrhage are frequent occurrences in advanced stage patients and represent independent characteristics unrelated to age or tumor size (4, 27). In a comparative analysis, Liu et al. (28) reported that all Xp11.2 RCCs exhibited an infiltrative growth pattern, whereas Zhu et al. (29) found that all 6 Xp11.2 RCCs appeared as well-defined masses. Altinok et al. (16) discovered that 73% of pediatric Xp11.2 RCCs were associated with thick, fibrous capsules, and Zhu et al. (29) described a capsule sign with a clear edge in all 9 cases. Within our study, Xp11.2 renal carcinoma showed invasive growth, involving both the renal medulla and cortex, presenting as a heterogeneous mass with cystic components, calcifications, and hemorrhages, and revealing a visible pseudocapsule. The density and degree of tumor enhancement may be related to the compression of vasculature by fibrous tissues (27). Following contrast administration, the lesions exhibited a mild, prolonged enhancement pattern (30, 31). In this case, Xp11.2 RCC showed heterogeneous enhancement, likely attributed to the uniform microvessel area within the tumor (32).

Studies focusing on FDG-PET for RCC are scarce, primarily due to challenges in distinguishing the radioactivity of FDG accumulated in renal tumors from the radioactivity of FDG excreted via the urinary system during physiological processes. Moreover, false-negative cases can result in low sensitivity for RCC detection. Nonetheless, evidence suggests that FDG-PET, utilizing SUV, may play a role in predicting the pathological grade of renal tumors (33). Xp11.2 RCC is associated with an increased incidence of tumor thrombosis, lymph node metastasis, and later stages of disease (26). PET/CT staging demonstrates higher sensitivity compared to other imaging modalities. The PET/CT manifestations ofXp11.2 RCC exhibit significant hypermetabolic activity and consistently present varying degrees of renal vascular invasion, lymph node involvement, or distant metastasis. Further observation of the similarities and differences between PET/CT manifestations of Xp11.2 RCC and other types of renal cancers is warranted, particularly as more cases are accumulated.

Several studies have extensively investigated and documented the potential applicability of radiotracers beyond FDG for the purpose of characterizing and staging RCC (34–37). Leveraging the marked expression of carbonic anhydrase IX (CAIX) observed in 95% of ccRCC, the anti-CAIX monoclonal antibody girentuximab emerges as a promising candidate for ccRCC detection. In a study by Hekman et al. (37), ^89^Zr-girentuximab PET/CT emerged as an invaluable diagnostic tool, capable of guiding clinical decision-making when faced with diagnostic uncertainties related to ccRCC suspicion. The upregulation of the receptor tyrosine kinase c-MET in RCC demonstrates a correlation with overall survival in cases of metastatic disease (38). A novel PET ligand, ^68^Ga-EMP-100, rooted in a c-Met binding peptide, has been developed. Mittlmeier et al. (35) demonstrated the feasibility of visualizing c-MET expression using ^68^Ga-EMP-100, enabling effective clinicopathological staging in cases of metastatic RCC. Recent case reports have showcased the efficacy of ^68^Ga/^18^F-PSMA PET/CT in restaging recurrent renal cancer post-nephrectomy (34, 36). However, there remains an absence of documented experiences regarding the imaging of Xp11.2 RCC with specific radiotracers within the current literature. Recent investigations have highlighted the notable therapeutic impact of vascular endothelial growth factor receptor-targeted agents and mammalian target of rapamycin inhibitors in the treatment of metastatic Xp11.2 RCC (15, 39). Notably, radionuclide-labeled vascular endothelial growth factor receptor-targeted agents hold the potential to provide insightful contributions to the characterization, staging, and assessment of treatment response for Xp11.2 RCC. Further advancements in molecular imaging technologies and the development of novel tracers hold great promise in advancing the diagnosis and management of RCC, ultimately contributing to better patient outcomes and overall disease management.

Differentiating Xp11.2 RCC from other subtypes of renal cancer is crucial. Distinguishing features of ccRCC, such as necrosis and hemorrhage, often correlate with lymph node metastasis and renal vein infiltration, similar to those found in Xp11.2 RCC (40, 41). However, ccRCC stands out as a hyper-vascular tumor, displaying significantly greater enhancement in the corticomedullary phase. In contrast, Xp11.2 RCC exhibits less enhancement than ccRCC. Notably, overlapping morphological features between Xp11.2 RCC and pRCC are observed, as pRCC also tends to appear hypo-vascular. A well-differentiated papillary RCC (pRCC) is characterized by homogeneity, small size, regular shape, low-level enhancement, and a peripheral location. On T2-weighted imaging, pRCC typically presents as a mass with homogeneous low signal intensity, likely due to cytoplasmic or interstitial histiocytic hemosiderin deposition in the tumor cells (42, 43). Additionally, pRCC is considered a low-grade malignant tumor with a low likelihood of retroperitoneal lymph node metastasis and the formation of renal vein cancer emboli. In contrast, chromophobe RCC often localize peripherally and manifest as well-defined, large, solid masses without necrosis or calcification, frequently diagnosed at an early stage (44, 45).

The treatment for Xp11.2 RCC remains ill-defined. Radical surgical excision stands as the primary therapy for early-stage cases (46–48). Regarding adjuvant treatment, there is still no research elucidating the most optimal or reliable approach for individual patients (49, 50). Chemotherapy, such as sunitinib, can also be considered (51). However, regardless of the applied treatment, Xp11.2 RCC demonstrates a poorer prognosis (19, 52). Lifelong follow-up should be implemented, incorporating medical history, physical examination, laboratory tests, and imaging data, for a more accurate assessment of the patient’s prognosis. According to the study, T stage at presentation is the sole factor associated with both progression-free survival and overall survival in patients with Xp11.2 RCC (50, 53).

## Conclusion

In conclusion, Xp11.2 RCC exhibits unique biological characteristics and is associated with an increased incidence of tumor thrombosis, lymph node metastasis, and advanced disease stages. Long-term follow-up is essential to monitor the likelihood of recurrence and metastasis. In this report, we present a case that may contribute to a better understanding of the disease for clinicians. ^18^F-FDG PET/CT examination can comprehensively visualize the lesion’s location and extent, providing a basis for clinical tumor staging and aiding in treatment monitoring and follow-up. To address the limitations of FDG, the utilization of specific tracers designed for RCC or tracers that are not excreted via the urinary system would be ideal. Further advancements in molecular imaging technologies and the development of novel tracers hold great promise in advancing the diagnosis and management of RCC, ultimately contributing to better patient outcomes and overall disease management.

## Data availability statement

The original contributions presented in the study are included in the article/supplementary material, further inquiries can be directed to the corresponding authors.

## Ethics statement

Written informed consent was obtained from the individual(s), and minor(s)’ legal guardian/next of kin, for the publication of any potentially identifiable images or data included in this article.

## Author contributions

WH: Writing – original draft, Writing – review & editing. YP: Writing – review & editing. YZ: Writing – review & editing. YQ: Writing – review & editing. YL: Writing – review & editing. AW: Writing – review & editing. LK: Writing – original draft, Writing – review & editing.
